# Betacellulin-Induced Beta Cell Proliferation and Regeneration Is Mediated by Activation of ErbB-1 and ErbB-2 Receptors

**DOI:** 10.1371/journal.pone.0023894

**Published:** 2011-08-29

**Authors:** Yoon Sin Oh, Seungjin Shin, Youn-Jung Lee, Eung Hwi Kim, Hee-Sook Jun

**Affiliations:** 1 Lee Gil Ya Cancer and Diabetes Institute, Gachon University of Medicine and Science, Incheon, Korea; 2 Northwestern University, Evanston, Illinois, United States of America; 3 College of Pharmacy, Gachon University of Medicine and Science, Incheon, Korea; University of Bremen, Germany

## Abstract

**Background:**

Betacellulin (BTC), a member of the epidermal growth factor family, is known to play an important role in regulating growth and differentiation of pancreatic beta cells. Growth-promoting actions of BTC are mediated by epidermal growth factor receptors (ErbBs), namely ErbB-1, ErbB-2, ErbB-3 and ErbB-4; however, the exact mechanism for beta cell proliferation has not been elucidated. Therefore, we investigated which ErbBs are involved and some molecular mechanisms by which BTC regulates beta cell proliferation.

**Methodology/Principal Findings:**

The expression of ErbB-1, ErbB-2, ErbB-3, and ErbB-4 mRNA was detected by RT-PCR in both a beta cell line (MIN-6 cells) and C57BL/6 mouse islets. Immunoprecipitation and western blotting analysis showed that BTC treatment of MIN-6 cells induced phosphorylation of only ErbB-1 and ErbB-2 among the four EGF receptors. BTC treatment resulted in DNA synthetic activity, cell cycle progression, and bromodeoxyuridine (BrdU)-positive staining. The proliferative effect was blocked by treatment with AG1478 or AG825, specific tyrosine kinase inhibitors of ErbB-1 and ErbB-2, respectively. BTC treatment increased mRNA and protein levels of insulin receptor substrate-2 (IRS-2), and this was blocked by the ErbB-1 and ErbB-2 inhibitors. Inhibition of IRS-2 by siRNA blocked cell cycle progression induced by BTC treatment. Streptozotocin-induced diabetic mice injected with a recombinant adenovirus expressing BTC and treated with AG1478 or AG825 showed reduced islet size, reduced numbers of BrdU-positive cells in the islets, and did not attain BTC-mediated remission of diabetes.

**Conclusions/Significance:**

These results suggest that BTC exerts proliferative activity on beta cells through the activation of ErbB-1 and ErbB-2 receptors, which may increase IRS-2 expression, contributing to the regeneration of beta cells.

## Introduction

Islet transplantation is currently the most promising treatment for type 1 diabetes, but there are side effects associated with the immunosuppressive agents and limitations resulting from a shortage of pancreas donors [1]. Therefore, generation of new beta cells either *in vitro* or *in vivo* is a high priority issue in diabetes treatment, and the identification of factors regulating the expansion of insulin-producing cells has potential importance for the treatment of diabetes.

Betacellulin (BTC), a member of the epidermal growth factor (EGF) family, was originally identified as a growth-promoting factor in the conditioned medium of a mouse pancreatic beta cell carcinoma (insulinoma) cell line [2]. BTC is proteolytically processed from a larger membrane-anchored 178-aminio acid precursor, and mature BTC is a 32-kDa glycoprotein of 80 amino acid [3]. BTC is synthesized in a wide range of tissues in the adult body and in a large number of cultured cells, including smooth muscle cells and epithelial cells. BTC mRNA is particularly highly expressed in the pancreas, liver, kidney and small intestine [4], [5]. In the pancreas, high expression of BTC mRNA suggests that BTC may have physiological role in the development and function of pancreas. Indeed, BTC is known to induce proliferation and differentiation of endocrine precursor cells in the pancreas. BTC, together with activin-A, can convert populations of exocrine AR42J rat pancreatic acinar cells into insulin-secreting cells [6] and mediate the proliferation of a fetal pancreatic epithelial cell line [7] as well as a rat insulinoma cell line [8]. Administration of a recombinant adenoviral vector expressing BTC (rAd-BTC) into streptozotocin (STZ)-induced diabetic mice restores normoglycemia [9]. As well, either ubiquitous [10] or beta cell-specific [11] BTC overexpression improves glucose metabolism in mice.

The effects of BTC are mediated by binding to one or more of four receptors in the tyrosine kinase family: ErbB-1/EGFR, ErbB-2/HER2/neu, ErbB-3, and ErbB-4/HER4 [3], [12]. BTC was found to bind all possible combinations of heterodimeric ErbB receptor as well as ErbB-1 and ErbB-4 homodimers, based on results using cell lines engineered to ectopically express pairwise combinations of ErbB receptors [3]. When activated by binding of a ligand, tyrosine residues on the ErbB receptors become phosphorylated followed by secondary messenger recruitment. The Ras- and Shc-activated mitogen-activated protein kinase (MAPK) pathway and the phosphoinositide 3-kinase (PI3K)-activated Akt pathway are the most important signaling networks of most ErbBs [13]. These receptor signaling pathways are critical for cell proliferation, migration, differentiation, cancer progression and apoptosis [14]. To understand the biological role of ErbB receptors on the pancreas, transgenic mouse models have been established and their phenotypes characterized, but most ErbB receptor-deficient mice die at embryonic day 10.5–13.5 and development of the islets is impaired [15]–[17]. These results indicate that ErbB receptors play an important role in the development of the pancreas.

Various transcription factors, forkhead box transcription factor O1, hypoxia-inducible factor-1α, and cAMP response element binding protein (CREB), have been implicated in BTC-mediated proliferation [18]–[20] and induce transcription of genes such as pancreatic and duodenal homeobox-1 and insulin receptor substrate (IRS)-2, which are known to be involved in beta cell proliferation [21], [22]. In this study, we investigated which ErbBs are involved in BTC-induced proliferation and regeneration of pancreatic beta cells and the mechanisms involved. We found that BTC activates ErbB-1 and ErbB-2 and consequently induces IRS-2 expression, contributing to beta cell proliferation and BTC-mediated beta cell regeneration in diabetic mice.

## Results

### Proliferative effect of BTC on beta cells

**Figure 1:**
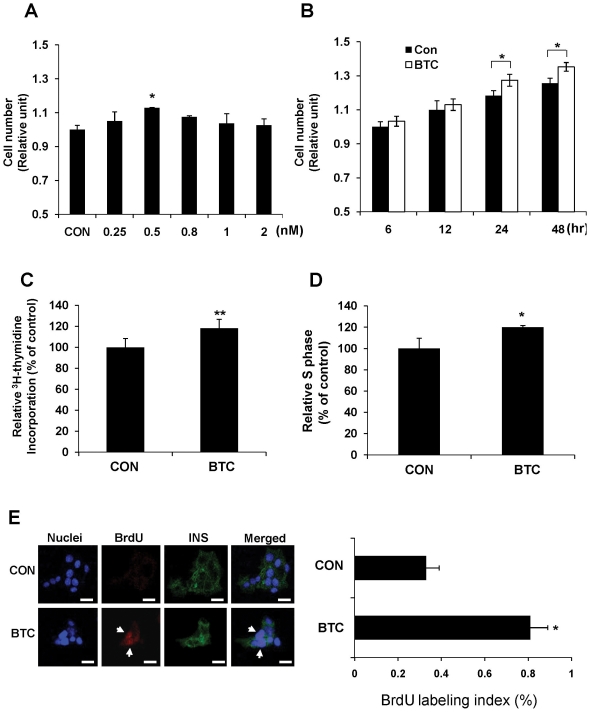
Proliferative effect of BTC on beta cells. The number of viable MIN-6 cells was determined after incubation with (A) various concentrations of BTC for 24 h or (B) 0.5 nM of BTC for various times. Relative cell numbers are expressed as fold changes over control levels (CON). (C) MIN-6 cells were incubated with 0.5 nM of BTC for 24 hrs and a ^3^H-thymidine incorporation assay was performed. The data are presented as the percent of untreated cells (CON). (D) Cells were harvested by trypsinization, stained with propidium iodide, and analyzed by flow cytometry. Values are percent of cells in S phase relative to that of untreated cells (CON). (E) MIN-6 cells were incubated without (CON) and with BTC (0.5 nM). Cells were fixed with paraformaldehyde and stained with DAPI (nucleus marker) and anti-insulin (INS) and anti-BrdU (BrdU) antibodies. The arrows show cells positive for insulin and BrdU. Proliferation of insulin-positive cells is shown as the number of BrdU/insulin double-positive cells as a percentage of the total number of insulin-positive cells (right panel). Values are means ± SEM of three experiments. Scale bar = 20 µm.*p<0.05, **p<0.01 vs CON.

To test the proliferative effect of BTC, a beta cell line (MIN-6 cells) was treated with various concentrations of BTC for 24 h and the cell viability was analyzed. We found that 0.5 nM of BTC (Fig. 1A) and incubation for 24 h (Fig. 1B) was optimal for the proliferative effects of BTC. To confirm the mitogenic effects, thymidine incorporation was measured at 24 h after stimulation with 0.5 nM BTC. Thymidine incorporation over 4 h was significantly increased in BTC-treated MIN-6 cells as compared with untreated cells (100% *vs* 118%, *p*<0.01) (Fig. 1C). The proportion of cells progressing to S phase, as analyzed by propidium iodide staining, was also higher in BTC-treated cells as compared with untreated cells (100% *vs* 120%, *p*<0.05) (Fig. 1D). 5-bromodexoyuridine (BrdU) incorporation into MIN-6 cells by BTC treatment, evidenced by an increase of insulin and BrdU double-stained cells as a percentage of insulin-positive cells (0.33% *vs* 0.81%, *p*<0.05), also confirmed the proliferative effect of BTC (Fig. 1E).

### Activation of ErbB-1 and ErbB-2 by BTC treatment in beta cells

**Figure 2:**
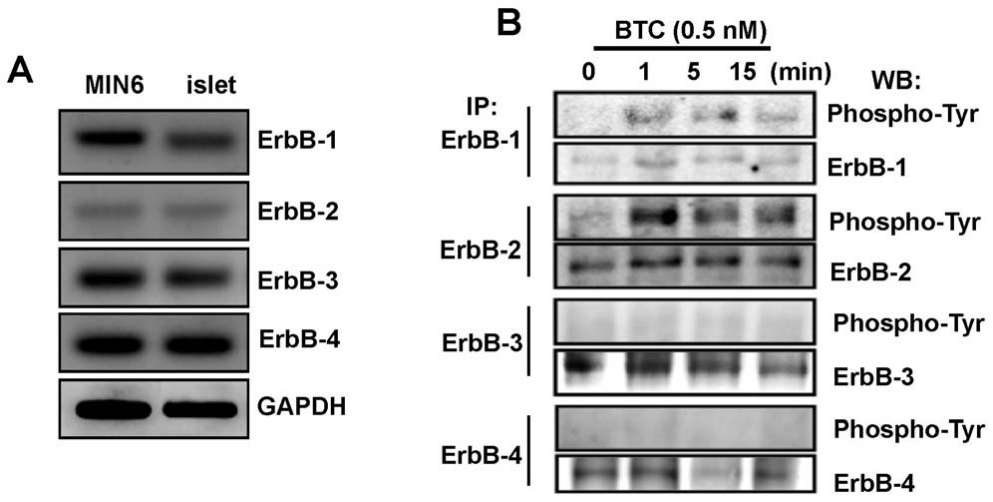
Expression of ErbBs and phosphorylation of ErbBs in MIN-6 cells treated with BTC. (A) mRNA expression of ErbBs in MIN-6 cells (MIN6) and isolated C57BL/6 mouse islets (islet) were determined by RT-PCR using specific primers. GAPDH was used as a loading control. (B) MIN-6 cells were incubated with BTC for the indicated times. Cell lysate was immunoprecipited (IP) with anti-ErbB antibodies and phosphorylated ErbBs were then detected by western blot (WB) using phospho-specific antibodies (Phospho-Tyr).

Because BTC exerts biological effects through ErbB receptors, we first examined the distribution of subtypes of ErbB receptors in islets isolated from C57BL/6 mice and in MIN-6 cells. RT-PCR analyses revealed that both MIN-6 cells and mouse islets express all four ErbB receptors, although ErbB-2 expression levels were lower than the other ErbBs (Fig. 2A). Next, we checked which EGF receptors were activated by BTC treatment. Cells were prepared after 0, 1, 5, or 15 min of incubation with BTC (0.5 nM), and phosphorylated ErbBs were examined by immunoprecipitation and western blot. As shown in Fig. 2B, phosphorylated ErbBs were barely detected in the absence of BTC treatment. Following addition of BTC, a striking increase in ErbB-1 and -2 phosphorylation was detected within 1 min, indicating that these two receptors were being activated by the BTC ligand. However, phosphorylation of ErbB-3 and ErbB-4 was not detected after BTC treatment.

### Inhibition of BTC-induced beta cell proliferation by ErbB-1 and ErbB-2 inhibitors

**Figure 3:**
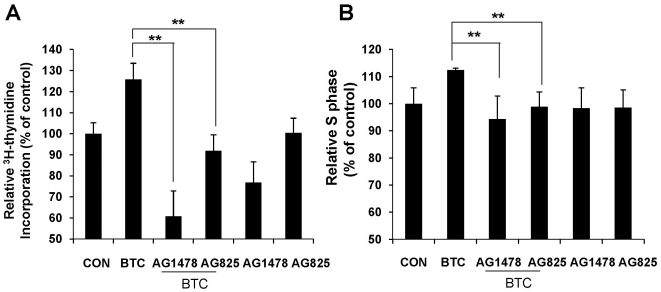
Inhibition of BTC-induced beta cell proliferation by ErbB-1 and ErbB-2 inhibitors. MIN-6 cells were incubated with BTC (0.5 nM) for 24 h with or without inhibitors AG1478 or AG825. (A) ^3^H-thymidine incorporation assay was performed. The data are presented as the percent of control (CON). (B) Cells were harvested by trypsinization, stained with propidium iodide, and analyzed by flow cytometry. Values are percent of cells in S phase relative to that of untreated cells (CON). Values are means ± SEM of three experiments, **p<0.01 vs BTC.

As we found that ErbB-1 and ErbB-2 were phosphorylated by BTC treatment, we examined whether inhibition of tyrosine phosphorylation of ErbB-1 and ErbB-2 would affect BTC-induced proliferation of beta cells. MIN-6 cells were stimulated with BTC (0.5 nM) for 24 h in the presence of 4 nM of AG1478 or AG825. AG1478 is known as a potent and selective inhibitor of ErbB-1 (IC_50_ = 3 nM), but does not inhibit ErbB-2 (IC_50_>100 µM) [23], and AG825 is a specific inhibitor of ErbB-2 [24]. Treatment of the cells with either inhibitor significantly suppressed BTC-induced DNA synthesis (Fig. 3A) and progression of G1 to S phase (Fig. 3B). These results suggest that the proliferative effect of BTC is mediated through ErbB-1 and ErbB-2 receptors.

### Inhibition of BTC-induced IRS-2 expression by ErbB-1 and ErbB-2 inhibitors

**Figure 4:**
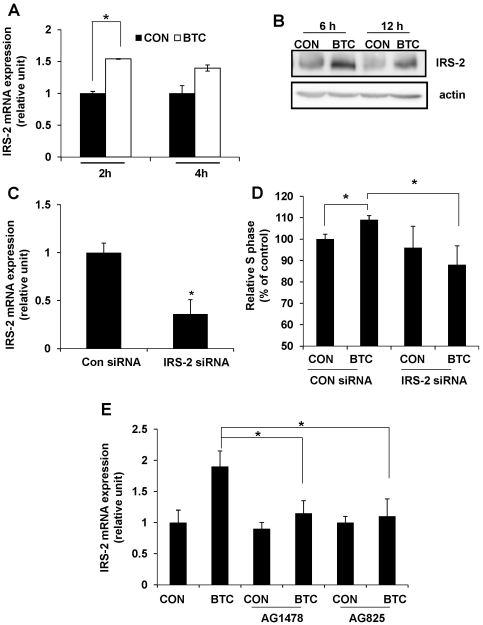
Inhibition of BTC-induced IRS-2 expression in beta cells. MIN-6 cells were incubated with BTC (0.5 nM) for the indicated times. (A) Total RNA was isolated and quantitative real-time PCR for IRS-2 mRNA was performed. (B) Total protein was isolated, and immunoblotting was done for IRS-2 or actin. (C) MIN-6 cells were transfected with IRS-2 siRNA or control (Con) siRNA. After 48 h, cells were harvested and mRNA expression of IRS-2 was analyzed by quantitative real-time PCR using IRS-2 primers. (D) After transfection with IRS-2-specific siRNA for 48 h, cells were treated with BTC for 24 h. Cells were harvested, stained with propidium iodide, and analyzed by flow cytometry. Values are percent of cells in S phase relative to that of untreated cells (CON). (E) MIN-6 cells were treated with 0.5 nM BTC in the presence or absence of ErbB inhibitors (4 nM) for 2 h. Total RNA was isolated and qRT-PCR for IRS-2 mRNA was performed. Values are means ± SEM of three experiments, *p<0.05.

Because IRS-2 is known to play a crucial role in beta cell survival and growth [25], [26], we investigated whether BTC regulates IRS-2 expression levels. We first tested effects of BTC on the expression of IRS-2 mRNA or protein. Treatment of MIN-6 cells with 0.5 nM of BTC significantly upregulated IRS-2 mRNA levels at 2 h of treatment (Fig. 4A). Protein levels of IRS-2 were also increased after 6 and 12 h of BTC treatment compared with the control (Fig. 4B). To determine whether inhibition of IRS-2 blocks the effect of BTC, we transfected cells with IRS-2 small interfering (si) RNA or a control siRNA and treated them with BTC. We found that IRS-2 expression (Fig. 4C) and the proportion of cells in S phase (109% *vs* 88%, *p*<0.05) (Fig. 4D) was significantly reduced in IRS-2 siRNA-transfected cells as compared with control siRNA-transfected cells. Next, we examined the effect of ErbB-1 and ErbB-2 inhibitors on IRS-2 expression, and found that treatment with AG1478 or AG825 completely blocked BTC-induced IRS-2 mRNA expression (Fig. 4E).

### Involvement of ErbB-1 and ErbB-2 receptors in BTC-induced regeneration in vivo

**Figure 5:**
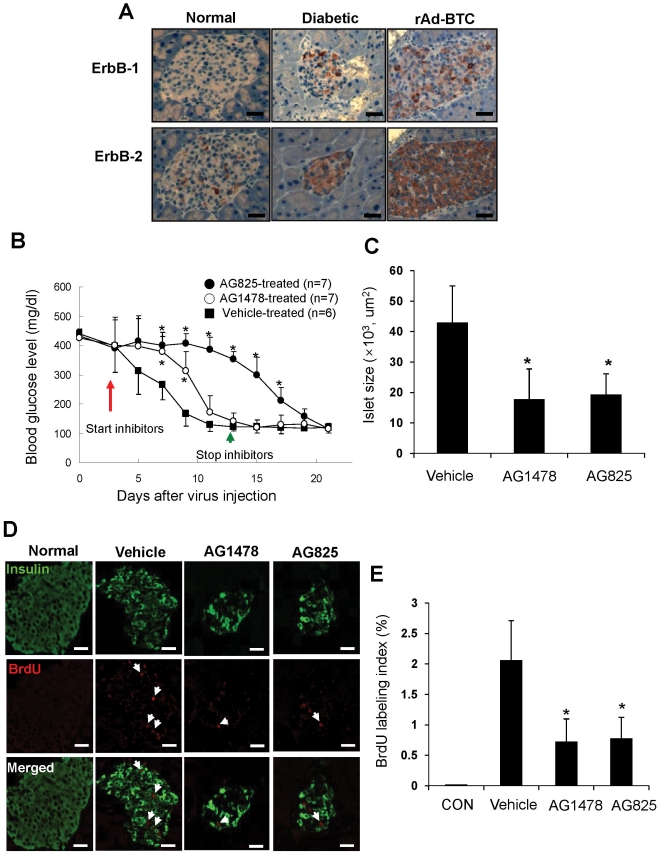
Involvement of ErbB-1 and ErbB-2 receptors in BTC-induced regeneration *in vivo*. (A) Pancreatic sections were prepared from untreated NOD.SCID mice (Normal), STZ-induced diabetic NOD.SCID mice (Diabetic), and STZ-induced diabetic NOD.SCID mice treated with rAd-BTC (rAd-BTC) and stained with anti-ErbB-1 or ErbB-2 antibodies. Photomicrographs of representative islets are shown. (B) STZ-induced diabetic NOD.SCID mice were treated with rAd-BTC (2×10^11^ particles). At 3 days after virus injection, mice were treated with vehicle, an ErbB-1 receptor inhibitor (AG1478) or an ErbB-2 receptor inhibitor (AG825, 500 µg in Captisol, i.p.) twice daily for 10 days. Blood glucose levels were measured. Values are mean ± SD of three experiments. (C) Mice were treated as in (B) and pancreatic sections were stained with hematoxylin and eosin. The total islet area was measured (n = 17 islets/group). (D) Mice were treated as in (B) and pancreatic sections were stained with anti-insulin and anti-BrdU antibodies. (E) Proliferation of insulin-positive cells is shown as the number of BrdU/insulin double-positive cells as a percentage of the total number of insulin-positive cells (right panel). Scale bars = 50 µm, Arrows indicate colocalization. n = 15 islets per group.*p<0.05 vs vehicle-treated group.

Previously, we observed that administration of a recombinant adenovirus expressing BTC (rAd-BTC) into streptozotocin (STZ)-induced NOD.SCID diabetic mice resulted in remission of hyperglycemia by regeneration of beta cells [9]. Since we found that ErbB-1 and ErbB-2 receptors are involved in BTC-mediated proliferation in MIN-6 cells, we investigated whether these receptors are involved in regeneration of pancreatic beta cells by BTC *in vivo*. We first checked the expression of ErbBs by immunohistochemical staining with anti-ErbB-1 and ErbB-2 antibodies in the pancreatic islets of normal mice, STZ-induced diabetic NOD.SCID mice, and STZ-induced diabetic NOD.SCID mice treated with rAd-BTC. Expression of ErbB-1 and ErbB-2 was detected in islets from STZ-induced diabetic mice and STZ-induced diabetic mice treated with rAd-BTC, but was barely detected in islets from normal mice. Interestingly, the upregulation of ErbB-2 expression appeared to be greater than the upregulation of ErbB-1 expression in the islets of diabetic mice (Fig. 5A).

Next, to examine whether ErbB-1 and ErbB-2 receptors are involved in BTC-induced remission of diabetes, we injected AG1478 or AG825 for 10 days into STZ-induced diabetic NOD.SCID mice treated with rAd-BTC beginning on the third day after virus injection and measured blood glucose levels. We found that injection of AG825 abrogated the remission of diabetes by rAd-BTC. However, when AG825 injections were stopped on the thirteenth day after virus injection, blood glucose levels gradually decreased and became normoglycemic (about 100 mg/dl) at 23 days after virus injection. The effect of AG1478 on the abrogation of BTC-induced remission of diabetes was less pronounced than that of AG825 (Fig. 5B). Islet size was significantly reduced in AG1478- or AG825-treated mice compared with mice injected with rAd-BTC alone (Fig. 5C).

To examine whether the decreased islet size in AG1478- or AG825-treated mice was caused by reduced beta cell proliferation, we injected rAd-BTC and inhibitors with BrdU, which labels dividing cells, for 5 days beginning on day 1 after rAd-BTC injection and stained pancreatic sections with anti-BrdU and anti-insulin antibodies. We found that double-stained cells were highly increased in islets of rAd-BTC-treated mice compared with normal mice, but the number of double-stained cells was significantly decreased by AG1478 or AG825 treatment (Fig. 5D, E).

## Discussion

The pancreatic beta cell mass is dynamic and can increase when exposed to appropriate environmental and physiological changes, such as obesity and pregnancy [27], and growth stimuli such as BTC, glucagon-like peptide-1 [28] and gastrin [29]. As diabetes results from absolute or relative deficiency of the beta cell mass, restoration of the beta cell mass might be one strategy for the treatment of diabetes. For this, various methods have been investigated including differentiation of insulin-producing cells from progenitor/stem cells and regeneration of beta cells.

We previously found that *in vivo* BTC expression by delivery of a recombinant adenovirus expressing BTC (rAd-BTC) remitted hyperglycemia in STZ-induced diabetic mice by beta cell regeneration [9], and some studies showed that new beta cells by BTC are generated mainly by replication of pre-existing beta cells [9], [30]. BTC is a potent growth factor that has positive effects on beta cell growth, through both increased proliferation and neogenesis. BTC promotes beta cell neogenesis from the pancreatic duct, therefore improving glycemic control after selective beta cell destruction with alloxan or after 90% pancreatectomy [31], [32]. In addition to BTC, other EGF receptor ligands have positive effects on beta cells. For example, EGF alone or combination with gastrin increased insulin secretion and beta cell numbers [33], and EGF treatment increased serum insulin levels and lowered blood glucose levels in diabetic mice [34]. Transforming growth factor-α increased beta cell growth and differentiation [35], and epiregulin, another EGF receptor ligand, stimulated beta cell proliferation and insulin release in pancreatic beta cell lines [36]. The functional redundancies of various EGF ligands suggest that EGF receptor binding with ligands via a conserved motif might be important mediators of beta cell mass regulation.

Because BTC produces physiological and pathological effects through its ErbB receptors, their distribution and activation are crucial for the function of BTC. It was reported that proliferative responses after BTC treatment were different between INS-1 cells and more primitive RINm5F cells, due to differential expression of ErbB family receptors [8]. Moreover, a BTC mutant protein, with a single mutation replacing glutamic acid with lysine, changed the binding affinity to ErbB receptors and resulted the differentiation of AR42J cells into insulin-secreting cells [37]. These studies indicate that the expression and affinity or activity of ErbBs play important roles for BTC function.

Sundaresan et al. reported that the ability of cells to respond to ligand is dependent on the expression of specific ErbB receptor combinations in the target tissue [7]. To investigate which ErbBs are involved in BTC-induced beta cell proliferation, we first checked the expression of ErbBs in MIN-6 insulinoma cells and mouse islets and observed that all known ErbB isoforms (ErbB-1, ErbB-2, ErbB-3, ErbB-4) were expressed both in MIN-6 cells and islets of C57BL/6 mice. Expression of four types of ErbB receptors in the MIN-6 cells suggests that multiple combinations of receptor heterodimers are able to form. However, we found that BTC induced only ErbB-1 and ErbB-2 phosphorylation in MIN-6 cells, suggesting that BTC may bind ErbB-1 and ErbB-2 in beta cells and deliver the signals for biological actions. Because it was reported that homodimers of ErbB-2 or ErbB-3 cannot mediate the growth factor signal and they form heterodimers with ErbB-1 and ErbB-4 [3], our results suggest that BTC-induced beta cell proliferation could occur through activation of ErbB-1 homodimers or ErbB-1/ErbB-2 heterodimer. As expected, BTC-induced DNA synthesis and cell cycle progression were suppressed by inhibitors of ErbB-1 or ErbB-2 receptors. These results clearly indicate that ErbB-1 and ErbB-2 activation might be involved in BTC action in MIN-6 cells, although it is not clear whether ErbB-1 homodimer or heterodimer of these receptors binds to BTC.

Signaling pathways mediated by ErbB-1 and ErbB-2 receptors involve the activation of Ras, MAPK, and the PI3K-activated Akt pathway, which activates several nuclear proteins, including cyclin D, a protein required for cell progression from G1 to S phase [13], [38]. We confirmed that cyclin D was translocated from cytosol to nucleus by BTC treatment (data not shown). Recently, many studies have shown that various transcription factors are involved in BTC-induced proliferation [13], [19]. One such transcription factor, CREB, leads to the expression of genes for beta cell function and survival and plays a role in mediating the effects of exendin-4, and BTC [20]. As IRS-2, an important mediator of beta cell proliferation, is one of the CREB-dependent genes [21], we confirmed that the expression of IRS-2 was upregulated by BTC treatment, and downregulation of IRS-2 blocked BTC-induced beta cell proliferation. These results indicate that IRS-2 expression is one mechanism by which BTC enhances beta cell proliferation, and this is mediated through ErbB-1 and ErbB-2 receptors.

We then asked whether ErbB-1 or ErbB-2 plays a role in the regeneration of beta cells and remission of diabetes in rAd-BTC-treated diabetic mice. We found an elevated expression of ErbB-1 and ErbB-2 protein in the pancreatic islets of STZ-induced diabetic mice or STZ-induced diabetic mice treated with rAd-BTC as compared with normal mice. Moreover, islet size and the number of proliferating beta cells in rAd-BTC treated mice was significantly reduced by treatment with ErbB-1 and ErbB-2 inhibitors. These results suggest that ErbB-1 and ErbB-2 expression in the islets of diabetic mice may facilitate BTC-induced regeneration of beta cells to compensate for beta cell loss. Once beta cells are destroyed, ErbB-1 and ErbB-2 expression may increase, and binding of these receptors to BTC may induce regeneration of beta cells.

Not only proliferation of pre-existing beta cells but also differentiation of beta cells from precursor cells residing in the pancreatic duct cells have been proposed as methods to increase beta cell mass [39], and BTC is known to have a role in these processes. A combination of hepatocyte growth factor and BTC-δ4 induced differentiation of pancreatic ductal epithelial cells into insulin-producing cells, and BTC gene transduction promoted beta cell differentiation from ductal cells [30], [40]. Kritzik et al. observed substantial expression of ErbB receptors in ductal cells of the regenerating pancreas, especially ErbB-2-positive cells were more prevalent than ErbB-3- or ErbB-4-positive cells [41]. Therefore, expression level of ErbBs might be relevant to ductal cell differentiation, which also contributes to beta cell regeneration.

Both ErbB-1 and ErbB-2 inhibitors abrogated the remission effect of BTC in diabetic mice, and the effect was most pronounced with the ErbB-2 inhibitor, which is correlated with the enhancement of ErbB-2 expression in islets of diabetic mice treated with rAd-BTC. It is not clear at the present time how the enhanced islet expression of ErbB-2 relates to beta cell regeneration. It was reported that expression of ErbB-2 was significantly induced in islets adjacent to areas infiltrated by immunocytes during interferon-γ mediated pancreatic regeneration [41]. Therefore, infiltrating immune cells might supply signals that mediate ErbB-2 induction in the regenerating islets. In addition, induction of ErbB-1 and ErbB-2 in our study might be part of a protective response elicited by the islet in response to the islet damage, but how this works for regeneration of beta cells requires further investigation.

In summary, our data shows that BTC activated ErbB-1 and ErbB-2 receptors in beta cells, deliver the signal, and consequently induce IRS-2 expression, which contributes to beta cell proliferation. *In vivo* studies also showed that ErbB-1 and ErbB-2 receptors are involved in BTC-induced beta cell regeneration at least in part by inducing beta cell proliferation.

## Materials and Methods

### Reagents

The sources of various reagents and materials were as follows: DNase I (Roche Diagnostics, Mannhein, Germany); polyclonal antibodies against ErbBs, secondary horseradish peroxidase-conjugated anti-mouse and anti-rabbit antibodies and anti-insulin antibody were from Santa Cruz Biotechnology Inc. (Santa Cruz, Ca, USA); anti-phosphotyrosine-20 antibody linked to horseradish peroxidase was from BD Transduction (Palo Alto, CA, USA); AG1478 and AG825 were from Calbiochem (La Jolla, CA, USA); anti-BrdU antibody was from Dako (Carpinteria, CA, USA); and recombinant mouse BTC was from R&D Systems (Minneapolis, MN, USA). All other biochemical reagents were from Sigma (St. Louis, MO, USA) or Invitrogen (Carlsbad, CA, USA).

### Cells

MIN-6 cells were routinely cultured in Dulbecco's modified Eagle's medium (DMEM) containing 25 mmol/l glucose supplemented with 15% (v/v) fetal bovine serum (FBS), 100 ug/ml streptomycin, 100 units/ml penicillin streptomycin, and 50 uM β-mercaptoethanol at 37°C in a CO_2_ humidified atmosphere. For experiments, cells were first incubated for 6 h in medium with 2% FBS-containing DMEM, and then incubated with various concentrations of BTC.

### Quantification of viable cells

MIN-6 cells were plated in a 96-well plate and incubated in the absence or presence of BTC. Viable cell number was checked by a CCK-8 assay kit (Dojindo Lab., Kumamoto, Japan) [42]. Briefly, 10 µl of the CCK-8 solution was added to each well, and the plate was incubated for 3 h. The absorbance of each well was measured at 450 nm using a microplate reader (Molecular Devices Corp., Menlo Park, USA). The experiment was repeated three times.

### ^3^H-Thymidine incorporation assay

For the measurement of DNA synthesis, 1 µCi/ml of ^3^H-thymidine (925GBq/mmol, Amersham) was added to each well for the last 4 h of culture. MIN-6 cells were then harvested with trypsin/EDTA and transferred on glass fiber filters using a cell harvester (Skatron Combi, Skatron, Norway). ^3^H-radioactivity was measured in a liquid scintillation counter. The experiment was repeated three times.

### Propidium iodide staining and flow cytometric analysis

After treatment with BTC or specific inhibitors, MIN-6 cells were harvested by treatment with trypsin-EDTA or centrifugation, respectively, washed twice with PBS, and fixed with ice-cold 70% ethanol. Cells were pelleted by centrifugation for 5 min at 3,000 rpm at 4°C, and the ethanol supernatants were discarded. Cells were then resuspended in a propidium iodide solution (50 µg/ml in PBS) with 50 µg/ml RNase A and incubated in the dark at room temperature for 20 min. The proportion of cells in S phase was then analyzed using flow cytometry (FACS Calibur; BD Transduction). The experiment was repeated five times.

### Immunofluorescence staining

MIN-6 cells were plated on 24-well culture plates with coverslips. After incubation with 0.5 nM of BTC for 24 h, the cells were washed three times with ice-cold PBS, and fixed in 4% paraformaldehyde in PBS for 15 min. Pancreata of BrdU-injected mice were isolated and embedded in paraffin. Pancreatic sections were deparaffinized in toluene, dehydrated in alcohol, and washed in water. After antigen unmasking, the slides were permeabilized in 0.5% Triton X-100, and non-specific protein binding sites were saturated with 2% BSA in PBS for 1 h. The cell and tissue sections were incubated with primary antibodies (rabbit anti-insulin, 1∶100; mouse anti-BrdU, 1∶100) overnight in a cold room and then washed three times with ice-cold PBS. Cells were incubated with fluorescein isothiocyanate-conjugated anti-rabbit or rhodamine-conjugated anti-mouse secondary antibodies for 30 min. Nuclei were then fluorescently labeled with DAPI. After washing four times with ice-cold PBS, the coverslips were mounted on glass slides with mounting medium. The labeled cells were observed under a confocal microscope (LSM 700, Carl Zeiss Inc., Oberkochen, West Germany) and BrdU-positive and insulin-positive cells were counted with Image Pro software (Media cybernetics, Inc., Bethesda, MA, USA). The BrdU labeling index was calculated as the number of BrdU/insulin double-positive cells as a percentage of the total number of insulin-positive cells (n = 15 islets/group).

### Isolation of islets from mice

Islets of Langerhans were isolated from C57BL/6 mice (12 weeks old) by the collagenase digestion technique. About 3 ml of a collagenase solution (0.8 mg/ml) was injected into the bile duct to inflate the pancreas. After the pancreas was removed, the tissue was incubated at 37°C for 5–10 min to complete the digestion. The islets were then separated by gradient centrifugation at 2000 *g* for 10 min on 25, 23, 21.5, and 11.5% Ficoll. Islets at the interface between the 21.5% and 11.5% Ficoll fractions were collected and washed with Hank's balanced salt solution. Healthy islets of appropriate size were hand-picked under a stereomicroscope. The dissociated islets were quickly frozen in liquid nitrogen for RNA isolation.

### Immunoprecipitation and immunoblotting

MIN-6 cells were incubated with 0.5 nM BTC for 0, 1, 5, or 15 min, and cell lysates were prepared. For immunoprecipitation, cell lysates (0.75 mg protein) were precleared by incubation with 30 µl resuspended Protein A/G PLUS beads and 1 µg normal rabbit IgG on a rotating platform for 1 h at 4°C, followed by centrifugation at 3,000 rpm for 5 min at 4°C. The supernatants were incubated with 2 µg anti-ErbB antibodies overnight at 4°C. Protein A/G PLUS beads were added to the lysate-antibody mix, followed by incubation for 1 h at 4°C. The beads were washed four times with lysis buffer. The immunoprecipitates of total cell lysates were resolved by SDS-PAGE, transferred onto polyvinylidenefluoride membranes, blocked for 1 h in 3% BSA in TBS-T (20 mM Tris-HCl, pH 7.5, 150 mM NaCl, 0.1% Tween 20) or 3% milk TBS-T, and incubated for 1 h with either anti-phospho-Tyr (1∶5000), anti-ErbB-1 (1∶100), anti-ErbB-2 (1∶500), anti-ErbB-3 (1∶500), anti-ErbB-4 (1∶500), or anti-IRS-2 (1∶1000) antibody. The blots were then incubated with horseradish peroxidase-conjugated anti-rabbit antibody. Signals were detected by using the ECL detection system (Pierce, Rockford, IL, USA)

### RT-PCR and real-time quantitative PCR

Isolation of total RNA was performed according to the manufacturer's recommended protocol for the Tri Reagent (Invitrogen), and extracted RNA was treated with DNase I and reverse transcribed to single-strand cDNA using oligo(dT) primer with PrimeScript™ RTase (Takara). Appropriate dilutions of each single-stranded cDNA were prepared for subsequent PCR amplification by monitoring the GAPDH gene as a quantitative control. Primer sequences were as follows:

5′-ATTCATGCGAAGACGTCACATT-3′ and 5′-GTTCCACGAGCTCTCTCTCTTGA-3′ for ErbB-1 (NM_207655; S, 2284–2305; AS, 2357–2335)

5′-GCTGCCCGAAACGTGCTA-3′ and 5′-CCGTGCCAGCCCGAA-3′ for ErbB-2 (NM_001003817; S, 2541–2559; AS, 2607–2592)

5′-AGGCTTGTCTGGATTCTGTGGTT-3′ and 5′-GGGATCGGGTGCAGAGAGA-3′ for ErbB-3 (NM_010153.1; S, 3122–3144 ; AS, 3194–3176)

5-GGAGGCTGCTCAGGACCAA-3′ and 5′-ACGCAGGCTCCACTGTCAT-3′ for ErbB-4 (NM_010154.1; S, 706–724 ; AS, 776–758)

PCR reaction was performed using the following conditions for ErbBs:

ErbB-1 and ErbB-2: 35 cycles of 1 min at 94°C, 1 min at 60°C and 1 min at 72°C

ErbB-3 and ErbB-4: 35 cycles of 1 min at 94°C, 1 min at 58°C and 1 min at 72°C

Samples were analyzed by gel electrophoresis and bands were revealed by staining gels with ethidium bromide. Real-time quantitative PCR analysis was performed using SYBR master mix (Applied Biosystems) using the ABI 7500 Real-time PCR system according to the protocols provided by the manufacturer (Applied Biosystems). IRS-2 was amplified using the forward primer 5′-CAGAGCAAGAACCTGACTGGTGTAT and the reverse primer 5′-GGCTGTTCGCAATTGAGCTT. The relative mRNA transcript levels were calculated according to the 2^−ΔCT^ method [43], in which ΔCT represents the difference in threshold cycle values between the target mRNA and the cyclophilin internal control. The experiment was repeated five times.

### Transfection with siRNA

The IRS-2 siRNAs were purchased from Dharmacon Research (Lafeyette, CO, USA) as siGENOME SMART pool. It targeted four regions of IRS-2 mRNA for interference. The target sequences were as follows: IRS-2 #1 GGACGAGUACUUCGCUGUA, #2 UCAUGUCCCUUGACGAGUA, #3 GUGAAAGAGUGAAGCGCUA, #4 ACUCGGUGG UGGCGCAGAA. A synthetic Cyclophilin B control pool siRNA was used as a negative control. MIN-6 cells (1×10^5^ cells/well) were plated in a 12-well plate, transfected with 5 pmole of siRNA and lipofectamine reagent in serum-free medium and incubated for 3 h at 37°C in a CO_2_ incubator. Following incubation, the cells were supplied with growth medium containing 15% fetal bovine serum.

### In vivo treatment with rAd-BTC and tyrosine kinase inhibitors

Recombinant adenoviral vectors expressing BTC (rAd-BTC) were constructed and produced as previously described [9]. NOD.SCID mice were made hyperglycemic by injection of STZ (100 mg/kg body weight in citrate buffer, pH 4.5, I.P. twice) and STZ-induced diabetic mice (blood glucose levels >400 mg/dl) were injected intravenously with rAd-BTC (2×10^11^ particles) through the tail vein [9]. A tyrosine kinase inhibitor, AG1478 or AG825 (500 µg), in 100 µl of 100 mM of Captisol (Cydex Inc.), an agent that improves the solubility, stability, and bioavailabilty of these inhibitors [44], was intraperitoneally injected twice daily for 10 days beginning on the third day after virus injection. Vehicle alone (100 mM Captisol) was injected as a control. Blood glucose levels were measured in the tail vein blood twice daily for 10 days using a glucometer (One Touch; Lifescan, Milpitas, CA, USA). All experiments using mice were approved by the Institutional Animal Care and Use Committee at the Rosalind Franklin University of Medicine and Science and Gachon University Medicine and Science (LCDI-2008-0021).

### Immunohistochemical analyses

Pancreata were fixed in either methacarn (60% methanol v/v, 30% chloroform v/v, and 10% glacial acetic acid v/v) or 10% buffered formalin overnight and embedded in paraffin, to be used for hematoxylin-eosin and immunohistochemical staining. Tissue sections were placed in an oven (95°C for 15 min, 10 mM citrate, pH 6.0) for antigen retrieval and blocked with blocking solution (5% goat or horse serum, 1% BSA and 0.05% Tween-20 in PBS). Tissues were then incubated with goat anti-ErbB-1 or rabbit anti-ErbB-2, -3, or -4 (Santa Cruz, 1∶100). HRP-conjugated goat anti-rabbit IgG (1∶500) or HRP-conjugated horse anti-goat IgG (1∶500) (Chemicon) were used as secondary antibodies, and peroxidase staining was performed with 3,3-diaminobenzidine (DAB) as a chromogen (Liquid DAB+ Substrate Chromogen System; Dako). The size of each islet (n = 17 islets/group) was measured in hematoxylin and eosin-stained slides using a reticule with a phase-contrast microscope (Carl Zeiss Inc.) and Axio Vision software.

### Statistical analysis

Data are presented as means ± SEM. Statistical analyses were performed using one-way factorial ANOVA. Pair-wise comparisons were evaluated by Duncan's multiple range test. *P*<0.05 was accepted as statistically significant.
